# Supplementary material for: Plasmonic imaging of living pancreatic beta-cell networks
Source: Sci Rep. 2026 Jan 3;16:3993. doi: 10.1038/s41598-025-34094-0 (PMC12855867; doi:10.1038/s41598-025-34094-0)
Supplement: Supplementary file 1 — Supplementary Material 1 [file 41598_2025_34094_MOESM1_ESM.docx]

## **Supplementary Information**

## **Patch-clamp validation of SPRM-detected oscillations in MIN6 beta-cells**

To provide evidence that the oscillations observed via Surface Plasmon Resonance Microscopy (SPRM) reflect underlying bioelectrical activity, we performed patch-clamp recordings on MIN6 cells under experimental conditions matching those used during SPRM.

**Materials and Methods**

The methods were similar to those previously published^1^ and are presented here in brief.

*Cell Culture –* MIN6 pancreatic beta-cells were cultured following previously established protocols. Cells were maintained in RPMI 1640 medium supplemented with 11 mM glucose, 10% fetal bovine serum (FBS), 10 mM HEPES, and 50 µg/mL penicillin-streptomycin. For patch-clamp experiments, cells were seeded onto glass coverslips and incubated for 24 hours prior to measurements under the standard tissue culture conditions described above.

*Solutions –* Pipettes were filled with a solution containing in mM: 140 KCl, 2.5 CaCl_2_, 1.2 MgCl_2_, and 10 HEPES (pH 7.4 with NaOH). The external bath solution was Hanks’ Balanced Salt Solution (HBSS). To stimulate electrical activity, 10 mM glucose was added. Nifedipine (10 µM final concentration) was applied from a 20 mM DMSO stock after glucose stimulation. Experiments were performed at 33 °C.

*Patch-Clamp Recording Protocol –* Recordings were performed using the cell-attached configuration, with pipettes held at 0 mV. Currents were recorded using an Axoclamp 1B amplifier (Molecular Devices), low-pass filtered at 500 Hz (8-pole Bessel), and digitized at 10 kHz using LabChart 8 (ADInstruments).

**Data Analysis**

*Spike detection* – Spikes were identified using a dynamic, threshold-based detector in MATLAB. Each recording segment (before/after drug application) was analyzed independently. The baseline was estimated as the median of the signal, and the noise level was estimated using the median absolute deviation (MAD) (robust σ). Spikes were defined as downward deflections exceeding baseline − 3 × noise, with a 50 ms refractory period to reduce false positives. Spike frequency was computed dynamically using non-overlapping 5-second windows, and the mean spike frequency was reported for each condition: HBSS (0 mM glucose, baseline), HBSS containing 10 mM glucose and HBSS containing 10 mM glucose and 10 µM nifedipine.

*Statistical comparison of interspike intervals* – To test the effect of nifedipine on firing dynamics, interspike interval (ISI) distributions under 10 mM glucose versus 10 mM glucose supplemented with nifedipine were compared using a two-sample Kolmogorov–Smirnov (KS) test for cells where both conditions contained ≥ 2 spikes. For cells where nifedipine completely suppressed spiking activity, this was recorded as complete functional inhibition without statistical comparisons. Significant differences (p < 0.05) were interpreted as evidence of altered calcium channel function.

**Results**

Three experimental conditions were tested on MIN6 cells: (1) HBSS alone (baseline), (2) HBSS containing 10 mM glucose, and (3) HBSS containing 10 mM glucose and 10 µM nifedipine. Figure S1 shows three representative cell recordings illustrating the effects of glucose and nifedipine on action current activity in MIN6 cells. In the absence of glucose (HBSS), cells displayed spontaneous electrical activity at a lower spike frequency. Glucose stimulation increased spike frequency, consistent with calcium-dependent electrical excitability. Nifedipine application reduced or abolished these oscillations, confirming the involvement of L-type calcium channels. In two cases, nifedipine fully suppressed spiking, precluding statistical comparison but clearly indicating functional inhibition.

Statistical analysis of ISI distributions provided quantitative evidence for nifedipine’s inhibitory effects. For Cell 1 (Fig. S1a), spikes were detected under both glucose and nifedipine. The KS test indicated a significant difference in ISI distributions *(p = 1.05 × 10⁻³)*, suggesting that nifedipine decreases firing dynamics. However, cell 2 (Fig. S1b) showed no detectable spikes under nifedipine treatment, making statistical comparison impossible. This complete suppression is considered a functionally meaningful indicator of calcium channel blockade. Cell 3 (Fig. S1c), under nifedipine treatment, showed significantly altered ISI distributions *(p = 1.03 × 10⁻³)*, indicating a decrease in firing behavior.


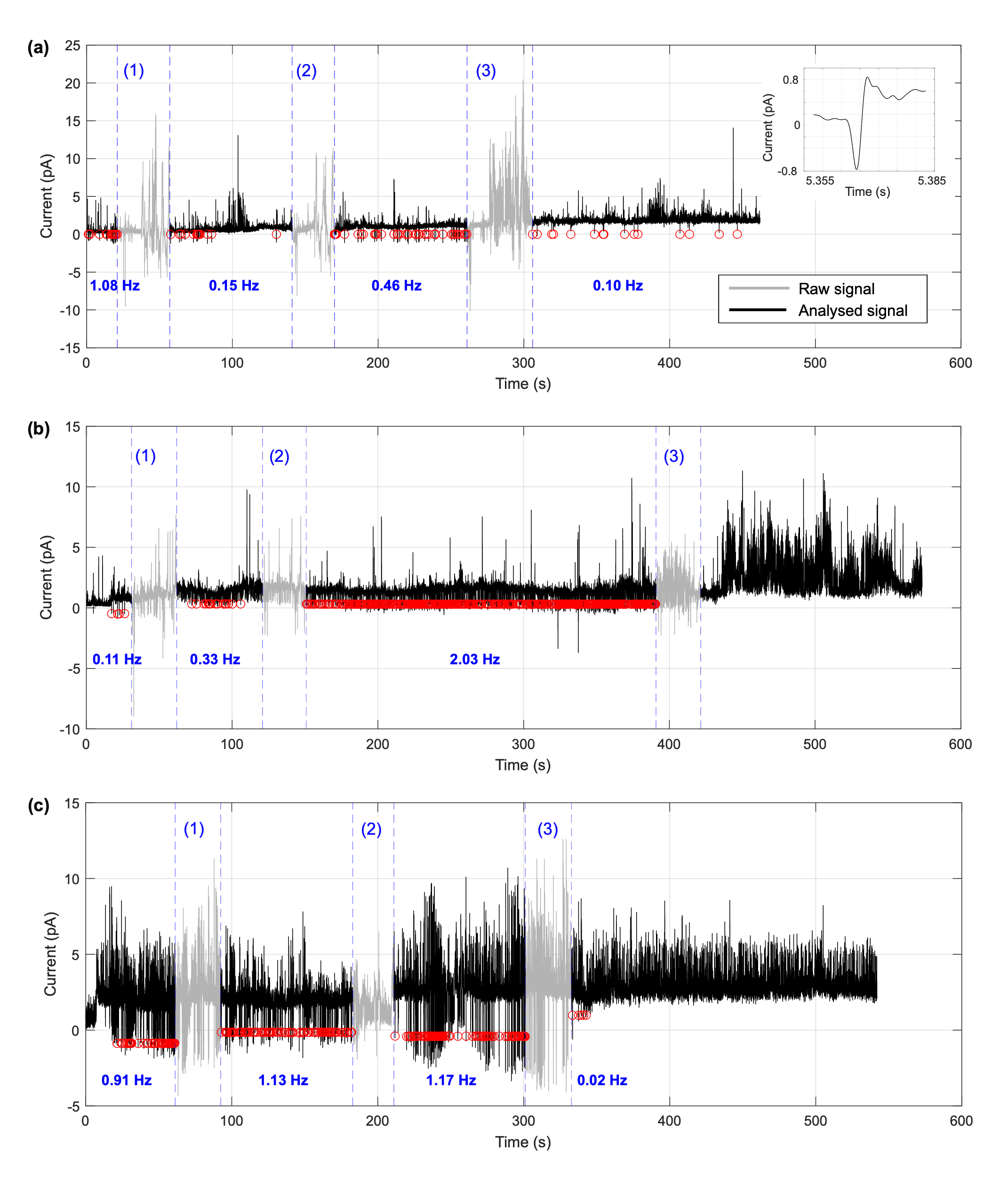


**Figure S1. Cell-attached patch-clamp of MIN6 under glucose and nifedipine exposure.** Representative cell-attached patch-clamp recordings of MIN6 cells from three experiments (in a, b, and c respectively) under three conditions: (1) HBSS without glucose (baseline), (2) HBSS with 10 mM glucose, and (3) HBSS with 10 mM glucose and 10 µM nifedipine. Two types of currents were recorded: upward positive deflections due to spontaneous activity of KATP potassium channels and downward negative deflection associated with action potential. The decrease in both amplitude and frequency of the KATP channels with glucose is a hallmark of pancreatic beta-cell electrophysiology^2^. Software-identified downward currents representing action currents are flagged with a circle. Glucose induces higher frequency oscillatory inward currents, which are suppressed upon nifedipine treatment, consistent with the activation and subsequent blockade of voltage-gated calcium channels characteristic for this cell type^2^. These findings support the interpretation that SPRM-detected oscillations arise from underlying bioelectrical signaling. Grey shaded regions in the traces represent background perfusion noise. In panels (a–c), Numbers indicate the averaged spike frequency for each condition (1–3), computed using non-overlapping 5-second windows

## **Correlation Analysis of High-Density SPRM Channels**


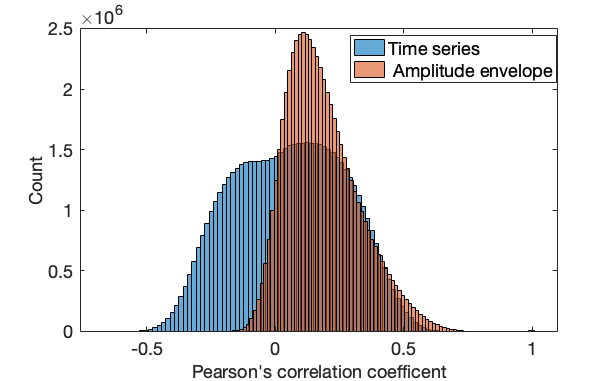


**a)**


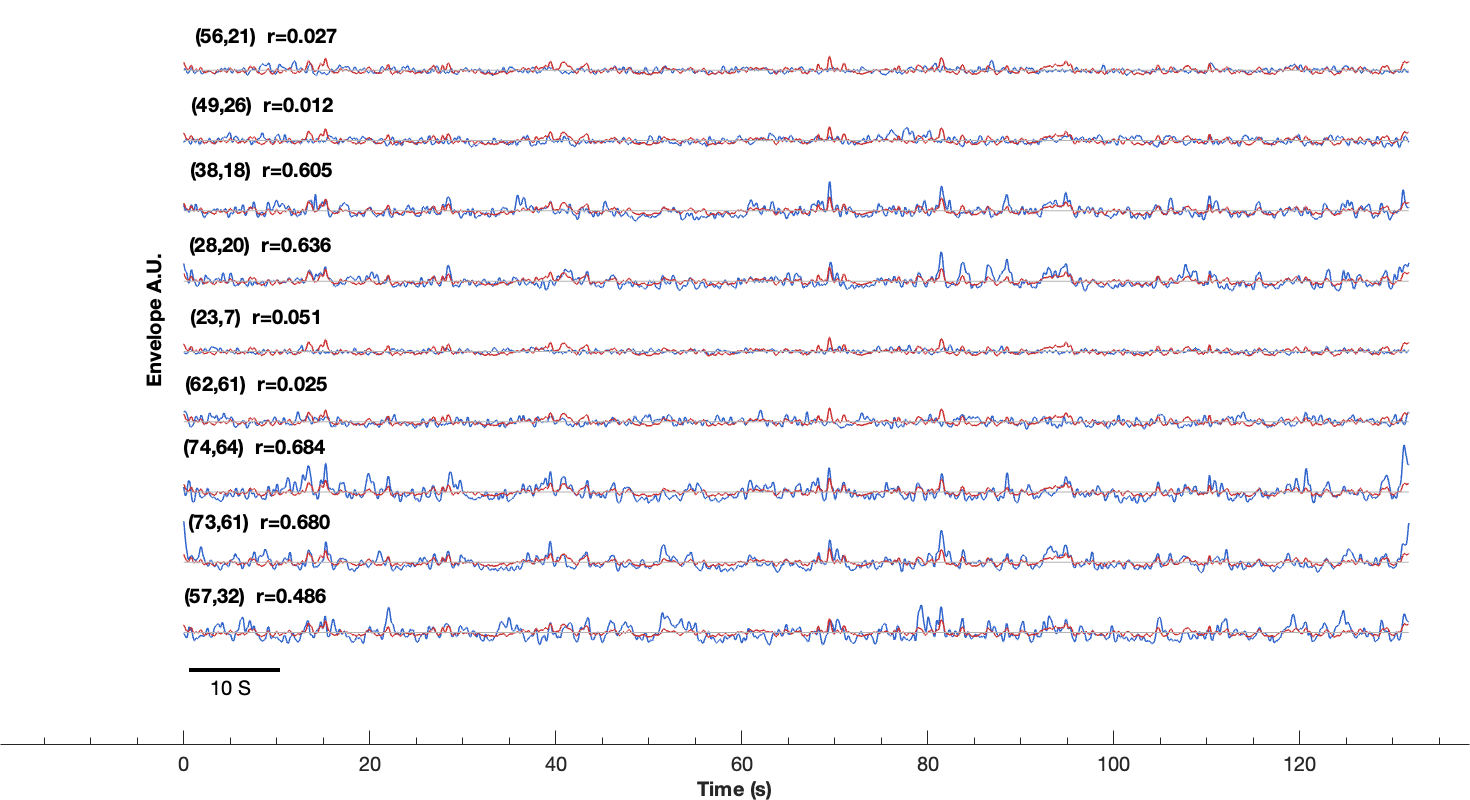


**c)**


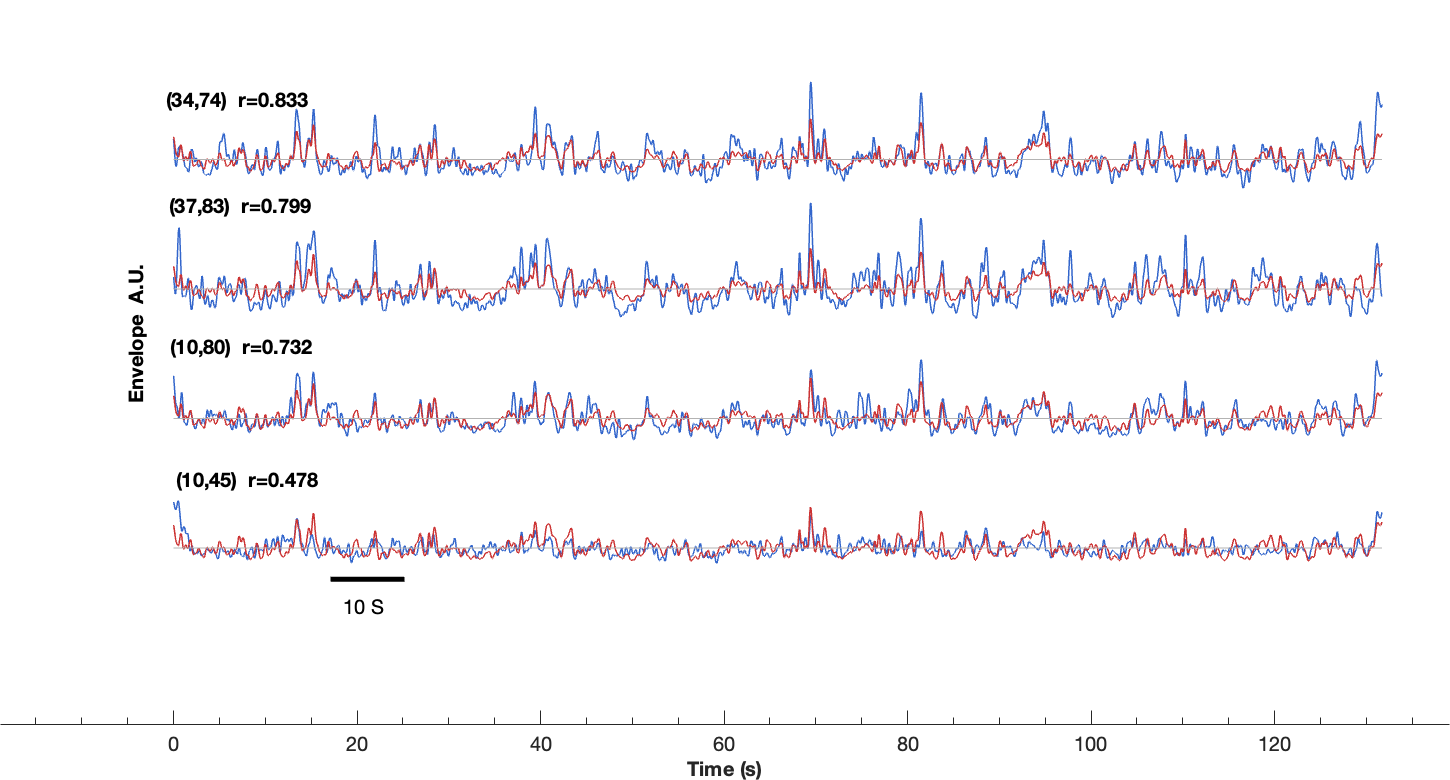


**b)**

***Figure S2* Validation of correlation statistics using the Hilbert transform.** a) Histogram of correlation coefficients before and after the Hilbert transform, demonstrating agreement in distribution, with differences arising from the Hilbert transform–extracted envelope being insensitive to phase variation. b) Example traces comparing an exemplar cell-level ROI (red) with extracellular background single pixel channels (blue). c) Examples of subcellular (blue) versus cell-level (red) ROI pairs showing varying correlation levels. All traces are Hilbert-transformed, and the instantaneous amplitude envelope was extracted and smoothed using a Gaussian window (width of 0.5 seconds) to reduce high-frequency fluctuations; ROIs correspond to channels shown in Fig. 4bi and Fig. 4bii of the main text. Pearson’s correlation coefficients are displayed on each plot.

**Fig. 5 Supplementary Information**


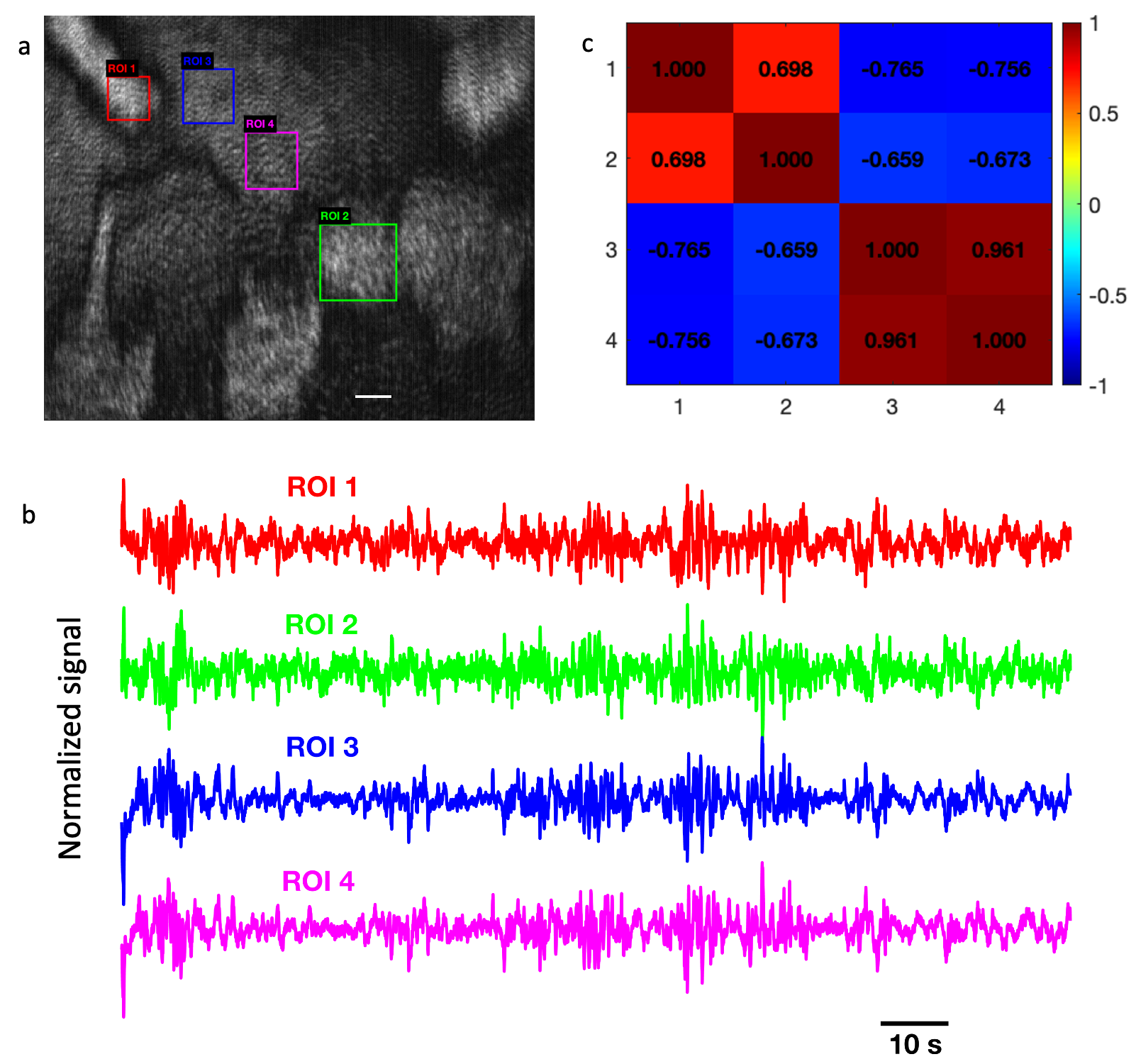


**Figure S 3. SPRM correlation patterns for cell and background ROIs – a**. SPRM image with labelled cell ROIs, scale bar 10 μm (1, 2) and background ROIs (3, 4). **b**. ROI traces filtered from 0.1–15 Hz using a 4th-order Butterworth filter. Sampling frequency is 100 Hz. **c.** Pearson cross-correlation showing background ROIs are highly correlated, similar to cell ROIs, while the two groups are anticorrelated due to the sensor’s transfer function.

**Saponin treatment to exclude illumination artefacts.**

**
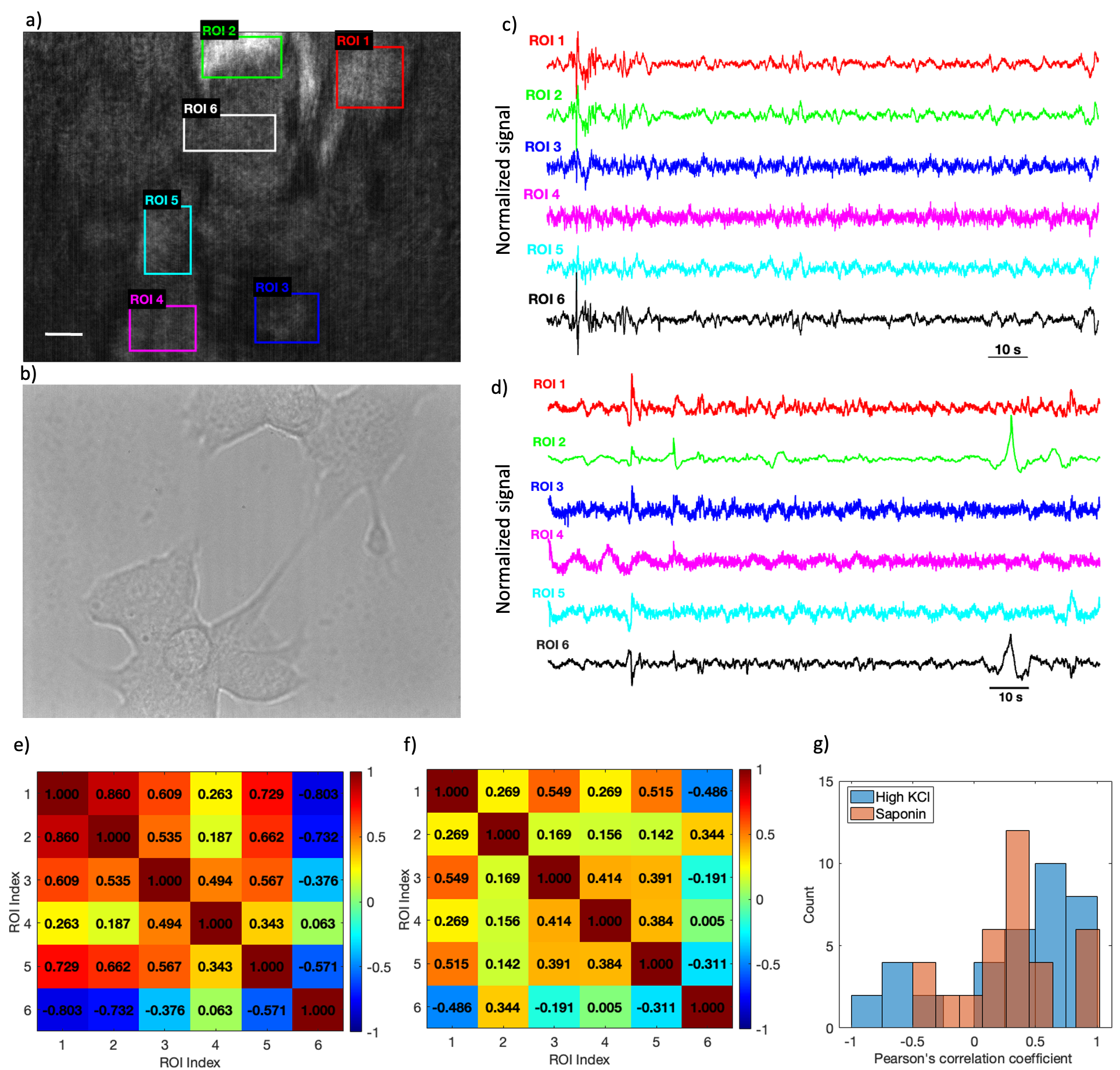
**

**Figure S 4. Effect of saponin treatment (**a) SPRM image. (b) Bright-field microscopy image. (c, d) Representative signal traces acquired in HBSS containing 50 mM KCl and in 0.1% saponin, respectively. Filter 4^th^ order Butterworth 0.1 – 15 Hz, sampling frequency of 100 Hz (e, f) Pearson correlation matrices with corresponding correlation distributions for data collected under the same conditions. (g) Correlation histograms before and after saponin treatment showing a drop in correlation between ROIs due to the lysing effect of saponin, as shown in Fig S5.

**
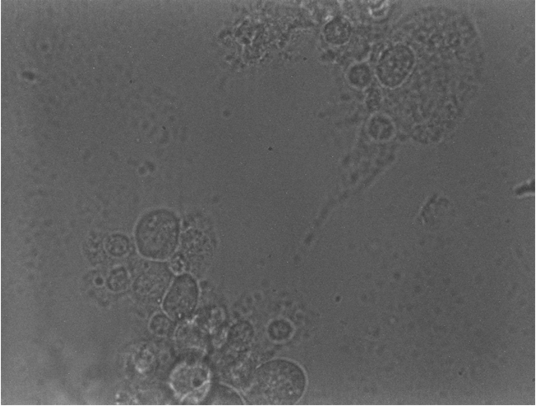
**

**Figure S 5 lysing effect of saponin treatment –** Bright field map of MIN6 after 0.1% saponin treatment.

**Photothermal effects**

SPRM was conducted using a fibre-coupled solid-state laser delivering 5 mW at the fibre input. After accounting for coupling and transmission losses through the high‑NA objective and intermediate optics, we estimate that approximately 1% of the input power reached the sample, corresponding to around 50 µW over a 150 µm (diameter) field of view, with an irradiance of roughly 0.3 W cm⁻².

Photothermal and photodynamic effects at 690 nm require the presence of strongly absorbing photothermal agents, such as porphyrins or nanoparticles^3^, to occur at intensities of 2–1.5 W cm⁻² and induce thermal responses. In contrast, our cells were not loaded with photothermal agents, and the irradiance is below the level at which photo-biomodulation studies report the onset of thermal effects in tissue in the 600–700 nm range (~300 mW cm⁻²)^4^. Furthermore, under these illumination conditions (red and far red), which are typically employed in live-cell microscopy to minimise phototoxicity, no alterations in cell morphology were observed.

**References**

1. Smith, P. A., Sellers, L. A. & Humphrey, P. P. Somatostatin activates two types of inwardly rectifying K+ channels in MIN-6 cells. *The Journal of physiology* **532**, 127 (2001).

2. Ashcroft, F. M. & Rorsman, P. Electrophysiology of the pancreatic β-cell. *Progress in biophysics and molecular biology* **54**, 87–143 (1989).

3. Lee, S., Min, S., Kim, G. & Lee, S. Recent advances in the design of organic photothermal agents for cancer treatment: A review. *Coordination Chemistry Reviews* **506**, 215719 (2024).

4. Zein, R., Selting, W. & Hamblin, M. R. Review of light parameters and photobiomodulation efficacy: dive into complexity. *Journal of biomedical optics* **23**, 120901–120901 (2018).
